# Supplementary material for: Unveiling the Bioactive Potential of the Invasive Jellyfish Phyllorhiza punctata Through Integrative Transcriptomic and Proteomic Analyses
Source: Biomolecules. 2025 Aug 4;15(8):1121. doi: 10.3390/biom15081121 (PMC12383608; doi:10.3390/biom15081121)
Supplement: Supplementary file 1 [file biomolecules-15-01121-s001.zip › Suplementary Material/FileS3/PEPFOLD4/XP_065072193_3953/clusters.html]

 PEP-FOLD clusters


Download archive of all models  here   

|  |  |  |
| --- | --- | --- |
| # | file | sOPEP |
| # cluster 1 - Size: 94 |  |  |
|  | XP\_065072193\_3953-model1 | -15.5083 |
|  | XP\_065072193\_3953-model1.1 | -15.5083 |
|  | XP\_065072193\_3953-model1.2 | -15.211 |
|  | XP\_065072193\_3953-model1.3 | -15.211 |
|  | XP\_065072193\_3953-model1.4 | -15.1775 |
|  | XP\_065072193\_3953-model1.5 | -15.1775 |
|  | XP\_065072193\_3953-model1.6 | -15.1775 |
|  | XP\_065072193\_3953-model1.7 | -15.1775 |
|  | XP\_065072193\_3953-model1.8 | -15.1209 |
|  | XP\_065072193\_3953-model1.9 | -15.1209 |
|  | XP\_065072193\_3953-model1.10 | -15.0445 |
|  | XP\_065072193\_3953-model1.11 | -15.0445 |
|  | XP\_065072193\_3953-model1.12 | -15.0445 |
|  | XP\_065072193\_3953-model1.13 | -15.0445 |
|  | XP\_065072193\_3953-model1.14 | -15.0188 |
|  | XP\_065072193\_3953-model1.15 | -14.9871 |
|  | XP\_065072193\_3953-model1.16 | -14.9491 |
|  | XP\_065072193\_3953-model1.17 | -14.91 |
|  | XP\_065072193\_3953-model1.18 | -14.8936 |
|  | XP\_065072193\_3953-model1.19 | -14.8046 |
|  | XP\_065072193\_3953-model1.20 | -14.8046 |
|  | XP\_065072193\_3953-model1.21 | -14.7808 |
|  | XP\_065072193\_3953-model1.22 | -14.7799 |
|  | XP\_065072193\_3953-model1.23 | -14.7635 |
|  | XP\_065072193\_3953-model1.24 | -14.7623 |
|  | XP\_065072193\_3953-model1.25 | -14.7618 |
|  | XP\_065072193\_3953-model1.26 | -14.7497 |
|  | XP\_065072193\_3953-model1.27 | -14.7497 |
|  | XP\_065072193\_3953-model1.28 | -14.7497 |
|  | XP\_065072193\_3953-model1.29 | -14.7127 |
|  | XP\_065072193\_3953-model1.30 | -14.6984 |
|  | XP\_065072193\_3953-model1.31 | -14.6865 |
|  | XP\_065072193\_3953-model1.32 | -14.6755 |
|  | XP\_065072193\_3953-model1.33 | -14.6755 |
|  | XP\_065072193\_3953-model1.34 | -14.6742 |
|  | XP\_065072193\_3953-model1.35 | -14.6738 |
|  | XP\_065072193\_3953-model1.36 | -14.6711 |
|  | XP\_065072193\_3953-model1.37 | -14.6619 |
|  | XP\_065072193\_3953-model1.38 | -14.6619 |
|  | XP\_065072193\_3953-model1.39 | -14.6619 |
|  | XP\_065072193\_3953-model1.40 | -14.6563 |
|  | XP\_065072193\_3953-model1.41 | -14.6563 |
|  | XP\_065072193\_3953-model1.42 | -14.6563 |
|  | XP\_065072193\_3953-model1.43 | -14.6563 |
|  | XP\_065072193\_3953-model1.44 | -14.6563 |
|  | XP\_065072193\_3953-model1.45 | -14.6563 |
|  | XP\_065072193\_3953-model1.46 | -14.6563 |
|  | XP\_065072193\_3953-model1.47 | -14.6563 |
|  | XP\_065072193\_3953-model1.48 | -14.6563 |
|  | XP\_065072193\_3953-model1.49 | -14.6442 |
|  | XP\_065072193\_3953-model1.50 | -14.6442 |
|  | XP\_065072193\_3953-model1.51 | -14.6442 |
|  | XP\_065072193\_3953-model1.52 | -14.6442 |
|  | XP\_065072193\_3953-model1.53 | -14.6442 |
|  | XP\_065072193\_3953-model1.54 | -14.6442 |
|  | XP\_065072193\_3953-model1.55 | -14.6442 |
|  | XP\_065072193\_3953-model1.56 | -14.6442 |
|  | XP\_065072193\_3953-model1.57 | -14.6442 |
|  | XP\_065072193\_3953-model1.58 | -14.6442 |
|  | XP\_065072193\_3953-model1.59 | -14.6148 |
|  | XP\_065072193\_3953-model1.60 | -14.6134 |
|  | XP\_065072193\_3953-model1.61 | -14.6093 |
|  | XP\_065072193\_3953-model1.62 | -14.5958 |
|  | XP\_065072193\_3953-model1.63 | -14.5958 |
|  | XP\_065072193\_3953-model1.64 | -14.5958 |
|  | XP\_065072193\_3953-model1.65 | -14.5934 |
|  | XP\_065072193\_3953-model1.66 | -14.5729 |
|  | XP\_065072193\_3953-model1.67 | -14.5681 |
|  | XP\_065072193\_3953-model1.68 | -14.5681 |
|  | XP\_065072193\_3953-model1.69 | -14.5677 |
|  | XP\_065072193\_3953-model1.70 | -14.5641 |
|  | XP\_065072193\_3953-model1.71 | -14.5641 |
|  | XP\_065072193\_3953-model1.72 | -14.5629 |
|  | XP\_065072193\_3953-model1.73 | -14.5604 |
|  | XP\_065072193\_3953-model1.74 | -14.5512 |
|  | XP\_065072193\_3953-model1.75 | -14.5291 |
|  | XP\_065072193\_3953-model1.76 | -14.5189 |
|  | XP\_065072193\_3953-model1.77 | -14.5146 |
|  | XP\_065072193\_3953-model1.78 | -14.5146 |
|  | XP\_065072193\_3953-model1.79 | -14.5146 |
|  | XP\_065072193\_3953-model1.80 | -14.5085 |
|  | XP\_065072193\_3953-model1.81 | -14.5012 |
|  | XP\_065072193\_3953-model1.82 | -14.4905 |
|  | XP\_065072193\_3953-model1.83 | -14.4905 |
|  | XP\_065072193\_3953-model1.84 | -14.4748 |
|  | XP\_065072193\_3953-model1.85 | -14.4628 |
|  | XP\_065072193\_3953-model1.86 | -14.4549 |
|  | XP\_065072193\_3953-model1.87 | -14.4529 |
|  | XP\_065072193\_3953-model1.88 | -14.4349 |
|  | XP\_065072193\_3953-model1.89 | -14.4027 |
|  | XP\_065072193\_3953-model1.90 | -14.3389 |
|  | XP\_065072193\_3953-model1.91 | -14.3046 |
|  | XP\_065072193\_3953-model1.92 | -14.1505 |
|  | XP\_065072193\_3953-model1.93 | -13.6484 |
| # cluster 2 - Size: 3 |  |  |
|  | XP\_065072193\_3953-model2 | -11.5054 |
|  | XP\_065072193\_3953-model2.1 | -11.4726 |
|  | XP\_065072193\_3953-model2.2 | -11.0779 |
| # cluster 3 - Size: 1 |  |  |
|  | XP\_065072193\_3953-model3 | -11.2223 |
| # cluster 4 - Size: 1 |  |  |
|  | XP\_065072193\_3953-model4 | -10.9103 |
| # cluster 5 - Size: 1 |  |  |
|  | XP\_065072193\_3953-model5 | -10.8933 |
|  |  |  |
